# Ontogenetic variation in the gut microbiota of *Kyphosus sydneyanus*: a comparative analysis

**DOI:** 10.1128/spectrum.02317-25

**Published:** 2025-10-20

**Authors:** Alessandro Pisaniello, Kim M. Handley, W. Lindsey White, Esther R. Angert, Kendall D. Clements

**Affiliations:** 1 School of Biological Sciences, University of Auckland, Auckland, New Zealand; 2 School of Science, Auckland University of Technology, Auckland, New Zealand; 3 Department of Microbiology, Cornell University 5922, Ithaca, New York, USA; Connecticut Agricultural Experiment Station, New Haven, Connecticut, USA

**Keywords:** gut microbiota, herbivory, ontogenetic variation, ddPCR, community assembly

## Abstract

Gut microbiota can be shaped by host-related (e.g., species, diet) and environmental (e.g., habitat, geography) factors. Gut microbiota of the herbivorous fish family Kyphosidae vary between individuals and gut sections and can be influenced by diet and geography. Temperate *Kyphosus sydneyanus* are abundant on rocky reefs of northeastern New Zealand, where adults mainly consume Phaeophyceae, whereas juveniles typically feed on Rhodophyta and Chlorophyceae. We compared the gut microbiota of *K. sydneyanus* adults and juveniles to investigate the relative effects of ontogeny versus diet on bacterial community composition. We used 16S rRNA gene amplicon sequencing and ddPCR quantification to determine the following: (i) ontogenetic variation in gut microbiota of *K. sydneyanus*, (ii) shared microbial taxa, and (iii) processes influencing bacterial community assembly, given the lack of vertical transmission. To further explore dietary effects, we also compared these data to the gut microbiota of adult *K. sectatrix*, which are rare tropical vagrants to northeastern New Zealand. Juvenile *K. sydneyanus* exhibited greater dietary similarity to *K. sectatrix* adults than conspecific adults. Overall, *K. sydneyanus* gut microbiota diversity increased with fish size, and *K. sydneyanus* adults had a more diverse gut microbiota than *K. sectatrix* adults. Estimated absolute abundances and community structure varied considerably across individuals. *K. sydneyanus* juveniles had relative abundances more similar to those from adult *K. sectatrix* than adult conspecifics. However, a comparison of amplicon sequence variants (ASVs) showed that juveniles of *K. sydneyanus* shared more ASVs with conspecific adults. This suggests that historical contingency and selection are important drivers of community assembly.

**IMPORTANCE:**

Most marine animals undergo external fertilization (e.g., fish) and lack mechanisms for vertical transmission of gut microbiota. Consequently, host-related and environmental factors can play important roles in community assembly. The gut microbiota of the herbivorous marine fish family Kyphosidae varies between individual fish, host species, diet, and geographic location. Juvenile *Kyphosus sydneyanus* shared more dietary similarity with adult *K. sectatrix* than adult conspecifics. Comparing gut communities of juvenile and adult *K. sydneyanus* and adult *K. sectatrix* collected from the same locations differentiates some of the causal factors involved in bacterial community assembly. Results suggest that the host diet has a strong influence on bacterial diversity and composition. In addition, historical contingency and environmental selection play a significant role in shaping gut microbiota through host ontogeny.

## INTRODUCTION

In general, community assembly is the process that determines the composition of communities of symbiotic gut microbes (1, 2). Fish gut microbiota communities can be shaped by both deterministic (predictable) and stochastic (random) forces associated with the surrounding environment (3–5). Overall, the assembly of gut communities is influenced by the habitat conditions (including host environment), presence/absence of microbial colonists, arrival succession, or chance-driven events that lead to community assembly (6). Conceptually, these processes can be summarized as environmental selection, dispersal limitation, historical contingency, and random sampling (6).

The relative proportions and taxonomic makeup of gut communities in fish are influenced by factors, including environment, seasonality, host diet, and ontogeny (7–11). The characterization of community composition in relation to these factors can be used to test hypotheses on the processes that drive microbial community assembly in fish hindgut systems. Community assembly processes are particularly important in the development of gut microbiota of fish during early ontogenetic stages (12) and are likely to play a continuing role as fish grow. Several studies have investigated the interactions of community assembly processes in aquaculture or farmed fish during monitored ontogenetic development (5, 13, 14). However, due to the mix of stochastic and deterministic events occurring in nature, little is known about how these processes (environmental selection, historical contingency, random sampling, and dispersal limitation [6]) influence community assembly in the gut microbiota of wild fish, especially marine herbivorous fishes where microbial metabolism contributes to host digestion (15) and vertical transmission is lacking (i.e., most species disperse gametes in the water column and lack parental care [8, 16]).

The most dominant phyla in the gut microbiota of marine herbivorous fish are *Bacillota*, *Bacteroidota,* and *Pseudomonadota* (formerly *Firmicutes*, *Bacteroidetes,* and *Proteobacteria*) (10, 17–20). Gut microbiota composition can vary within herbivorous fish species (10, 11), and establishing the functional importance of taxonomic composition can be difficult. However, different microbial taxa can play key roles in the digestion and health of some herbivorous fishes by degrading refractory carbohydrates and contributing to fish nutrition (21). Moran et al. (8) demonstrated that ontogenetic development in *K. sydneyanus* is associated with increased microbial diversity and short-chain fatty acid (SCFA) levels in adult fish (8). Host development also influences microbiota composition and community assembly in the gut of the lake sturgeon *Acipenser fulvescens* (4). Previous work investigated the influence of geographic location (10) and temporal (seasonal) variation (10, 11) on herbivorous fish in the family Kyphosidae. Here, we investigate the influence of ontogenetic development and dietary changes on community assembly processes in the gut of these fish.

The family Kyphosidae is distributed worldwide, and several species inhabit the tropical and temperate waters of Australasia (22). Tropical *Kyphosus* species from the Australian Great Barrier Reef (i.e., *K. vaigiensis* and *K. cinerascens*) harbor distinct and highly variable gut microbiota (10). *K. vaigiensis*, which has a diet dominated by Chlorophyta and Phaeophyceae, has a high proportion of the phylum *Spirochaetota* (formerly *Spirochaetes*)*,* while *K. cinerascens*, with a diet dominated by a mixture of Rhodophyta, Phaeophyceae, and Chlorophyta, has a high abundance of the phylum *Pseudomonadota* (10). In addition, gut bacterial communities in both tropical fish species vary greatly among individual fish, geographic locations, and along the hindgut (10). Gut communities in adults of the temperate *K. sydneyanus* in the Hauraki Gulf of New Zealand are similarly dominated by *Bacillota* and *Bacteroidota*, and at lower taxonomic levels (i.e., genus) vary among individual fish, radial gut location (mucosa versus lumen), and position along the intestine (11, 19, 20).

In northeastern New Zealand, *K. sydneyanus* juveniles and adults are abundant on rocky reefs, where adult individuals of the tropical species *K. sectatrix* are uncommon (23, 24). *K. sydneyanus* undergoes a dietary shift from a high proportion of Rhodophyta and Chlorophyta in juveniles to a diet of predominantly Phaeophyceae in adults (25). *K. sectatrix* in New Zealand was formerly referred to as *K. bigibbus*, although both are now considered distinct species (24). Adult individuals of *K. sectatrix* in different locations have been reported to have a diet with an equivalent proportion of both phaeophytes and rhodophytes (26), a higher abundance of rhodophytes (27), and a higher abundance of phaeophytes (28). The *K. sectatrix* adults found on rocky reefs in northeastern New Zealand are exposed to the same dietary algae and potential colonizing gut microbes as *K. sydneyanus* juveniles. However, *K. sectatrix* is also likely to require hindgut fermentation to extract energy from metabolites of phaeophytes such as mannitol and laminarin, as occurs in the gut of adult *K. sydneyanus* (21). The high variability in gut microbiota composition in *Kyphosus* species, the occurrence of ontogenetic diet shifts, and the influence of diet on gut microbiota composition (9, 10) raise the following questions: (i) how does the taxonomic composition of gut microbiota in *K. sydneyanus* respond to ontogenetic changes in diet, and (ii) is the gut microbiota of *K. sydneyanus* juveniles more similar to that of adult *K. sydneyanus*, which differ in diet from juveniles, or *K. sectatrix*, a different host species but which varies in diet compared to adult *K. sydneyanus*?

To answer these questions and to determine processes influencing community assembly in *Kyphosus* species, we examined the relative abundance, estimated the absolute abundance (using ddPCR) and the diversity of the gut microbiota in *K. sydneyanus* juveniles, and compared results with the microbiota of adult *K. sydneyanus* and *K. sectatrix*. We hypothesized that community assembly of gut microbiota in these fish is likely to be influenced by historical contingency and environmental selection, for example, that the order in which bacterial taxa colonize the gut may be influenced by ontogenetic changes in diet. Additionally, we compared the gut microbiota of warm temperate *K. sydneyanus* and *K. sectatrix* from north-eastern New Zealand with the gut microbial communities of the tropical species *K. cinerascens* and *K. vaigiensis* from the Great Barrier Reef, Australia (10). We hypothesized that dispersal limitation may play a fundamental role in shaping the gut bacterial community structure; that is, the pool of bacteria living in the environment may be a major driver of community assembly processes in these *Kyphosus* species.

## RESULTS

This study was conducted to examine how host-related and environmental factors influence gut microbiota community assembly in wild marine herbivorous fish (family Kyphosidae). These wild fish represent natural systems where the environment is not controlled, and in the study species, vertical transmission of gut microbiota is absent. To determine factors influencing community assembly, we analyzed the gut microbial communities in relation to host diet, bacterial diversity and composition (in relation to ontogeny and fish species), and environment/habitat (New Zealand temperate waters and Australian tropical waters).

### Host diet

**Fig 1:**
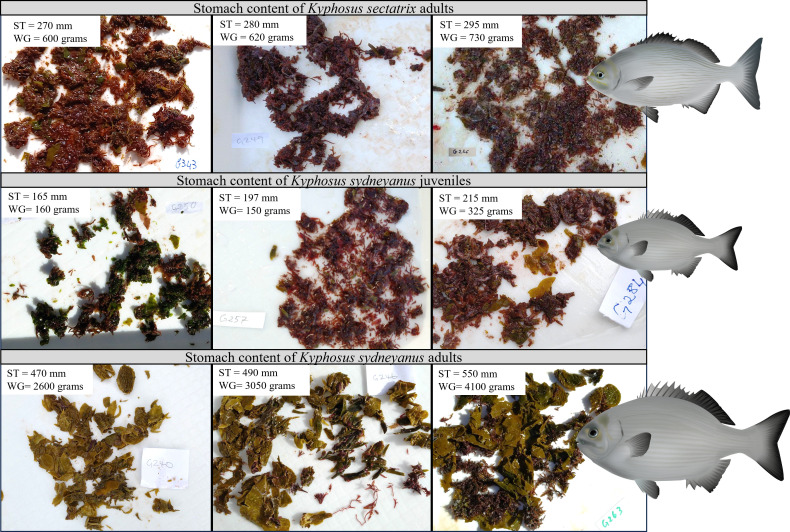
Stomach content photographs of seaweed ingested by individual fish of *Kyphosus sectatrix* (*n* = 3), *K. sydneyanus* juveniles (*n* = 3), and *K. sydneyanus* adults (*n* = 3). Stomach contents are dominated by rhodophytes in *K. sectatrix* adults and *K. sydneyanus* juveniles and phaeophytes in *K. sydneyanus* adults. ST, standard length of fish; WG, weight of gutted fish.

The stomach contents of all *K. sectatrix* and juvenile *K. sydneyanus* collected for the study were dominated by rhodophytes, with smaller proportions of phaeophytes (Fig. 1).

### Bacterial diversity varies with ontogeny and fish species

**Fig 2:**
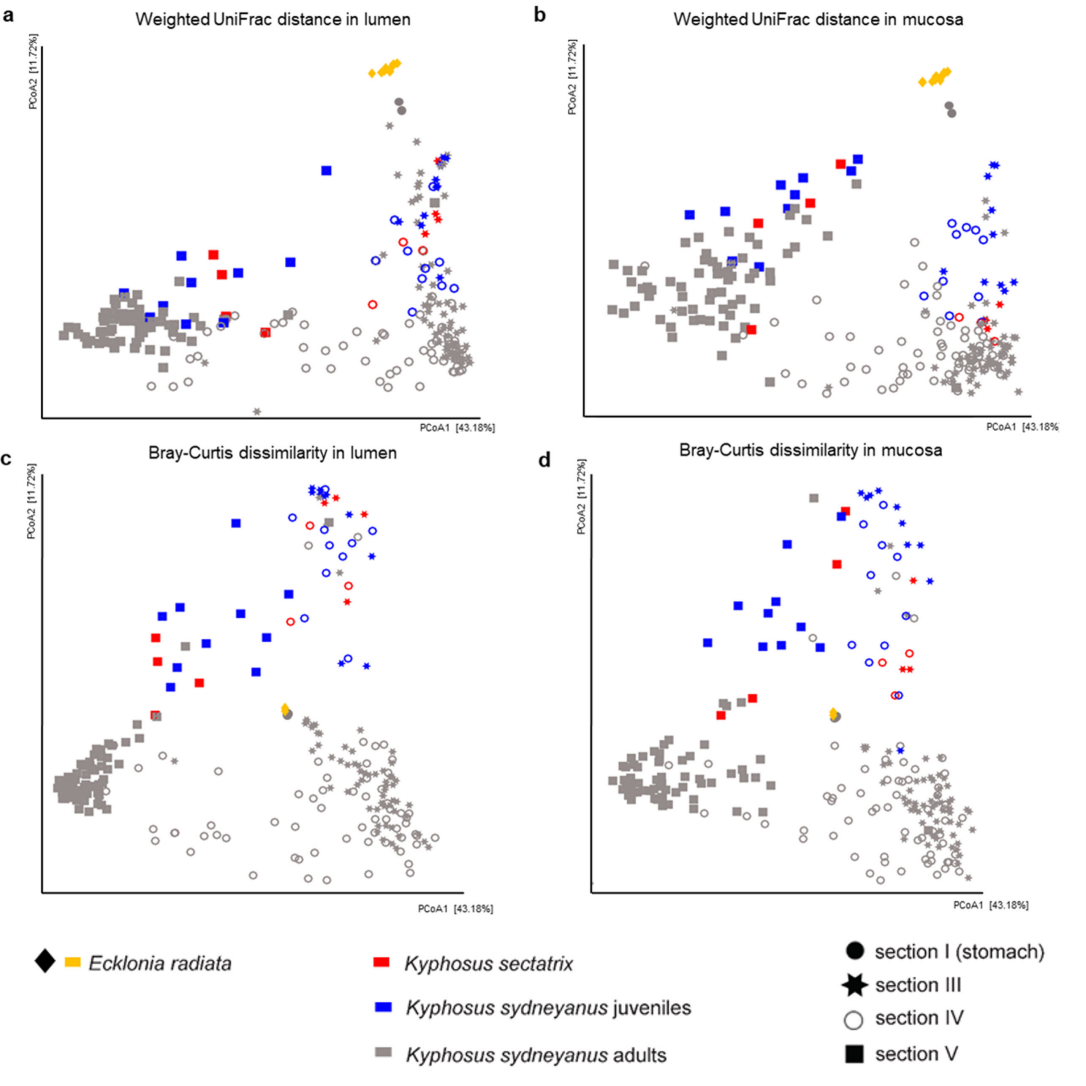
Bacterial community beta diversity as Principal Coordinate analysis (PCoA). (**a**) PCoA analysis of weighted UniFrac distance between bacterial communities from *Ecklonia radiata* (Phaeophyta), stomach contents, and luminal contents of gut sections of fish sample types; (**b**) PCoA analysis of weighted UniFrac distance between communities from *E. radiata*, stomach contents, and mucosal samples from gut sections of fish sample types; (**c**) PCoA analysis of Bray-Curtis dissimilarity distance between bacterial communities from *E. radiata*, stomach contents, and luminal contents of gut sections of fish sample types; (**d**) PCoA analysis of Bray-Curtis dissimilarity distance between communities from *E. radiata*, stomach contents, and mucosal samples from gut sections of fish sample types.

Overall bacterial diversity varied with fish species (*K. sydneyanus* and *K. sectatrix*) and ontogeny (*K. sydneyanus* adults and juveniles) in both gut locations (lumen and mucosa) and gut sections III, IV, and V (PERMANOVA < 0.001) (Fig. 2; Table S2). Weighted UniFrac distance, a measure of beta-diversity that quantifies the relative abundance and relatedness of microbial taxa between samples, showed that microbial beta diversity also differed between fish species and the microbiota of the phaeophyte *E. radiata* (PERMANOVA < 0.001, Table S2). The *E. radiata* bacterial community appeared more similar to the microbial community of section I (stomach content) of *K. sydneyanus* adults (Fig. 2a and b), which was dominated by phaeophytes (Fig. 1). Gut microbiota of *K. sydneyanus* juveniles and adult *K. sectatrix* were more similar to each other than the gut community of adult *K. sydneyanus*. Bray-Curtis dissimilarity, which quantifies the relative abundance of microbial taxa shared between samples (compositional level), showed similar trends as the weighted UniFrac PCoA (Fig. 2c and d). *E. radiata* and section I samples were located together in the PCoA plot, indicating close similarity between the communities in the phaeophyte samples and those from the stomach content of the fish (Fig. 2c and d).

**Fig 3:**
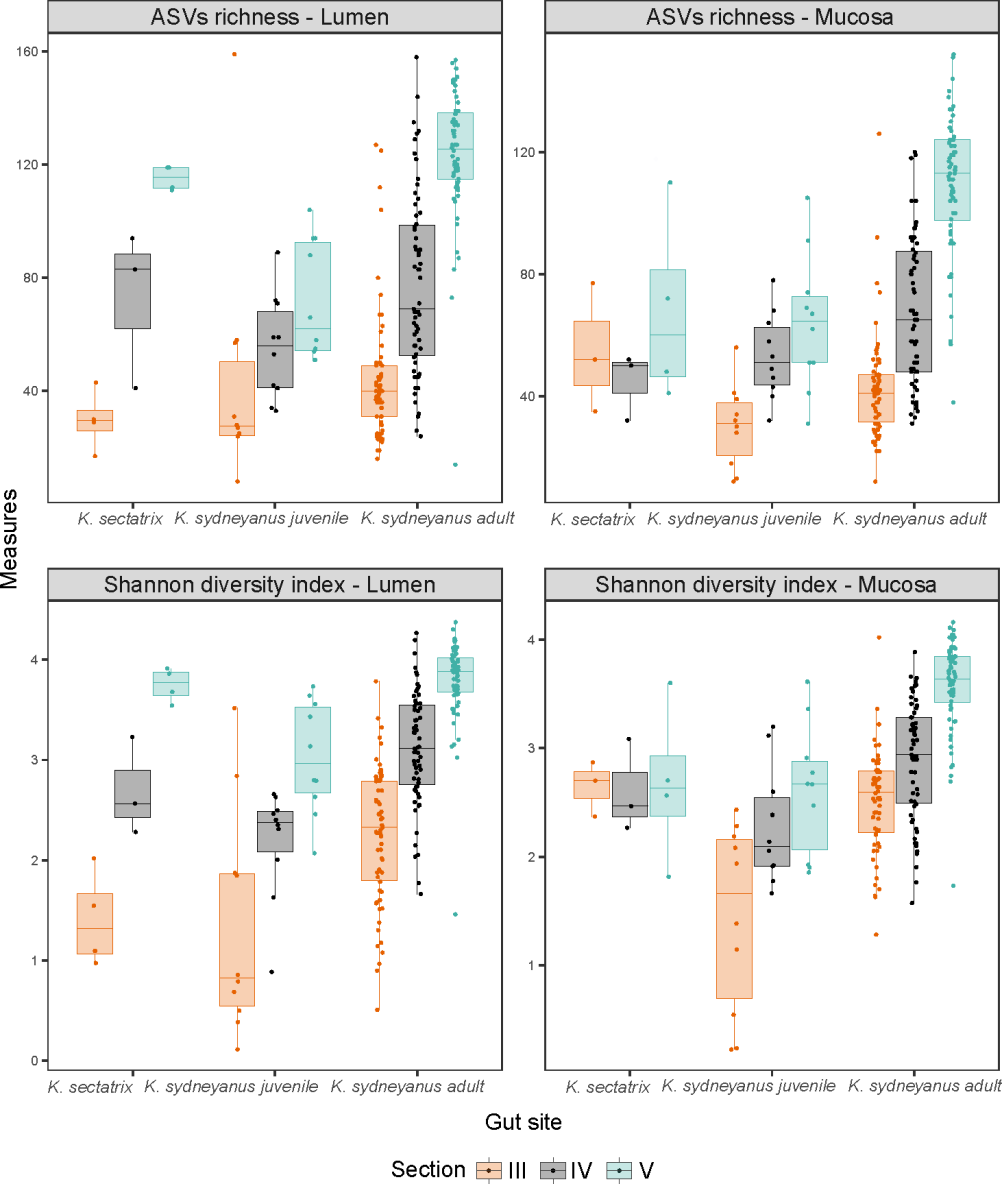
Boxplots of alpha diversity indices in *K. sectatrix*, *K. sydneyanus* juveniles, and *K. sydneyanus* adults across gut sections and gut locations (lumen and mucosa). Top boxplots show ASV richness indices; bottom boxplots show Shannon diversity indices.

Alpha diversity indices (amplicon sequence variant [ASV] richness and Shannon) also differed between fish species, age classes (*K. sydneyanus* juveniles and adults), hindgut sections (III, IV, and V), and gut sites (lumen and mucosa) (Fig. 3; Tables S3 to S6). Diversity in all lumen samples increased from gut section III to section V in all the samples (KW, Dunn post-hoc results were *P* < 0.01 for *K. sectatrix* adults*, P* < 0.01 for *K. sydneyanus* juveniles, and *P* < 0.001 for *K. sydneyanus* adults, Fig. 3). Mucosa samples showed similar trends to lumen samples from *K. sydneyanus* adults and juveniles, while in *K. sectatrix,* the hindgut sections of mucosa samples did not differ in either ASV richness or Shannon index. When comparing fish species, *K. sydneyanus* adults had the highest ASV diversity, followed by *K. sectatrix* adults and *K. sydneyanus* juveniles (Fig. 3).

### Bacterial microbiota composition of the different *Kyphosus* species

**Fig 4:**
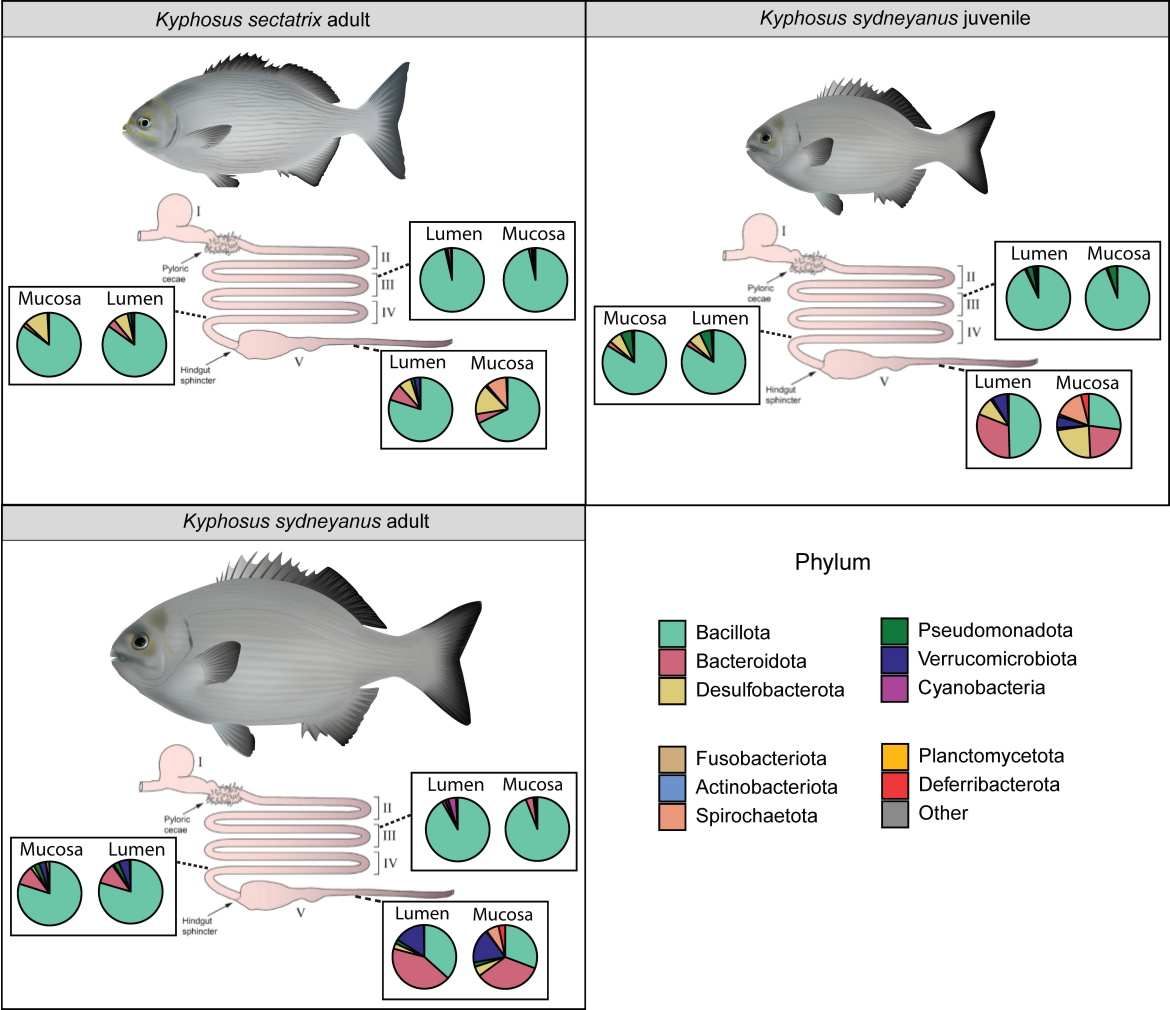
Mean relative read abundance by phylum level of hindgut sections III, IV, and V of both lumen and mucosa in *K. sectatrix* (*n* = 4 fish), *K. sydneyanus* juveniles (*n* = 10 fish), and *K. sydneyanus* adults (*n* = 64 fish).

In general, microbial communities were dominated by the phylum *Bacillota* in both lumen and mucosa samples of hindgut sections III and IV (Fig. 4). Section V displayed the greatest variation between fish sample types and gut locations (lumen and mucosa) (Fig. 4). In *K. sectatrix* section V, both lumen and mucosa were dominated by *Bacillota*, although *Desulfobacterota* and *Spirochaetota* were also relatively abundant in mucosa samples (Fig. 4). In *K. sydneyanus* juveniles, the section V lumen was likewise dominated by *Bacillota*, with the next most abundant phyla being *Bacteroidota*, *Desulfobacterota,* and *Verrucomicrobiota*. The section V mucosa communities in *K. sydneyanus* juveniles displayed a similar proportion of *Bacillota*, *Bacteroidota*, *Desulfobacteroidota,* and *Spirochaetota*, with *Verrucomicrobiota* and *Deferribacteroidota* both present at lower abundance. In *K. sydneyanus* adults, the microbial communities of both lumen and mucosa section V were dominated by *Bacteroidota* followed by *Bacillota* and *Verrucomicrobiota* (Fig. 4).

**Fig 5:**
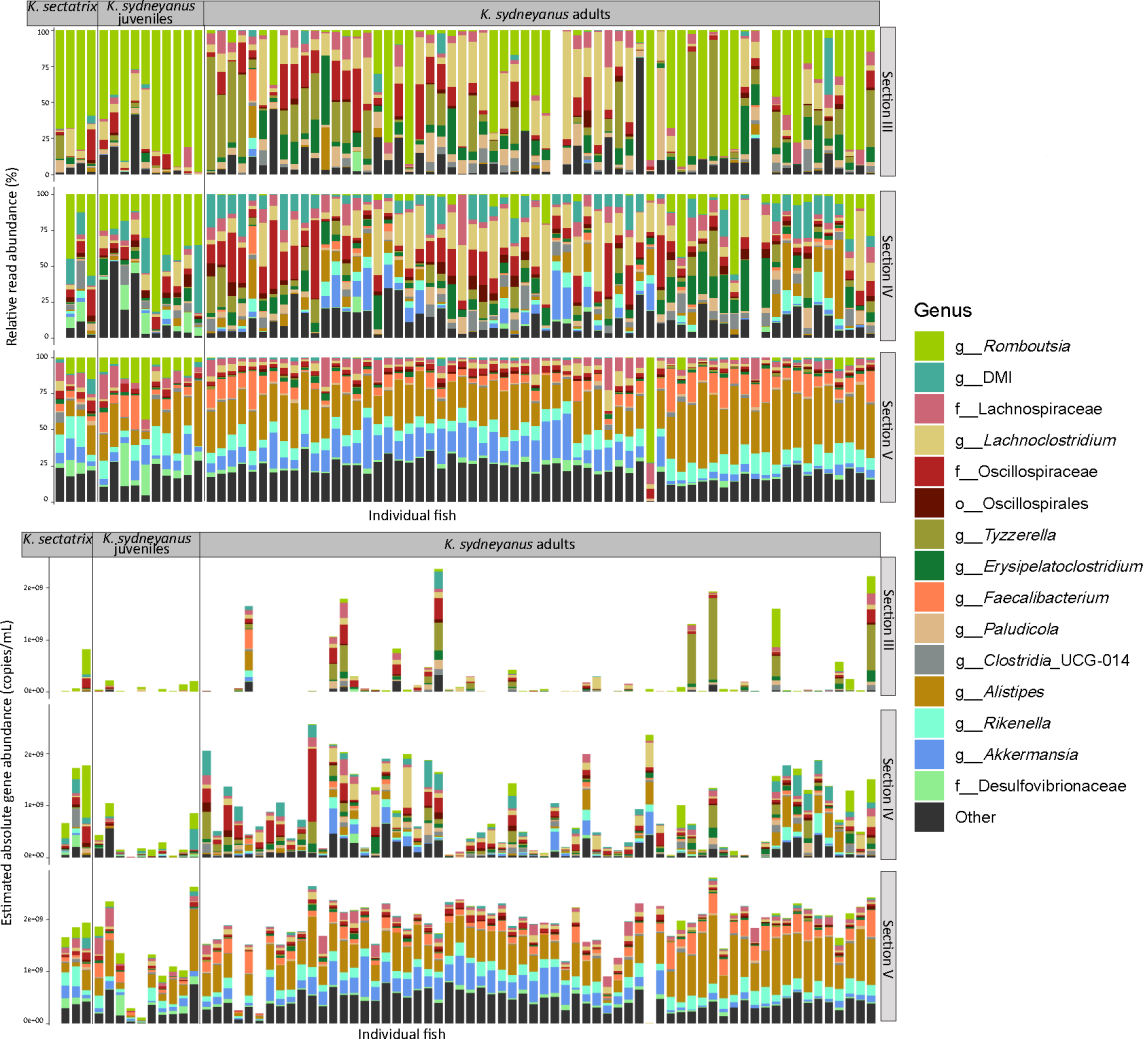
Top 15 genera of relative (top) and estimated absolute (bottom) read abundance of *K. sectatrix, K. sydneyanus* juveniles and *K. sydneyanus* adults across gut sections in the lumen.

**Fig 6:**
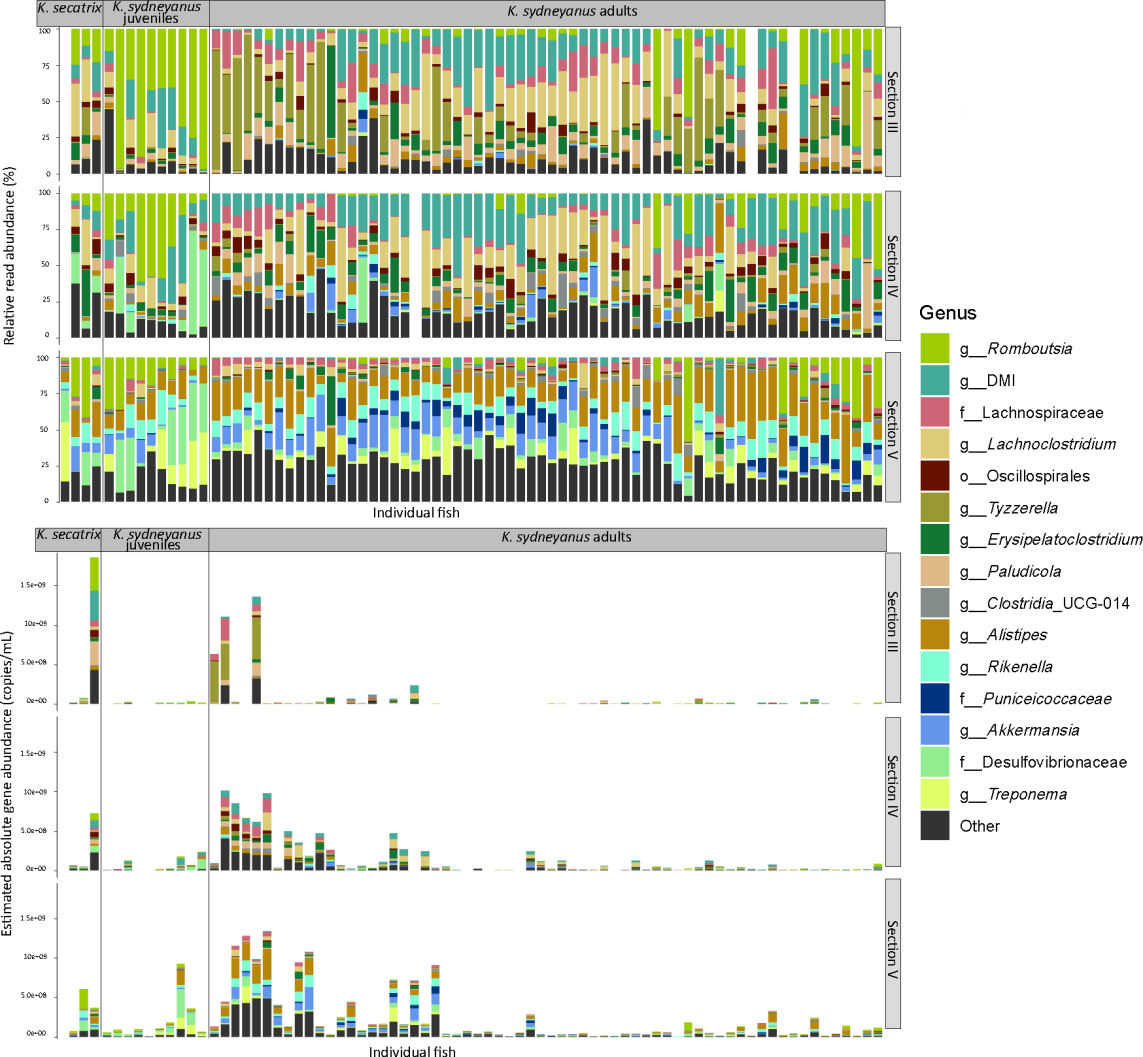
Top 15 genera of relative (top) and estimated absolute (bottom) read abundance of *K. sectatrix*, *K. sydneyanus* juveniles and *K. sydneyanus* adults across gut sections in mucosa.

The bacterial communities from the three fish sample groups (*K. sydneyanus* juveniles and adults, and *K. sectatrix*) were highly variable in terms of both composition at the genus level (% relative read abundance) and estimated absolute abundance (copies/mL) in lumen (Fig. 5) and mucosa (Fig. 6) samples. Overall, the gut microbial communities of *K. sectatrix* and juvenile *K. sydneyanus* appeared very similar in composition and structure. *Romboutsia* dominated section III of both fish types. The genus *Lachnoclostridium* was also moderately abundant in section III of some individuals of *K. sydneyanus* juveniles. The bacterial communities of *K. sydneyanus* adults in section III were dominated by *Rombutsia*, *Lachnoclostridium,* or *Tyzzerella,* varying in relative abundance across individual fish. Section IV varied greatly among host individuals and fish species, with candidate genus DMI (family *Acholeplasmataceae*) being abundant across some individual fish in all three sample groups. The genus *Rombutsia* was again dominant in *K. sectatrix* and juveniles of *K. sydneyanus,* while *Oscillospiraceae* and *Lachnoclostridium* were highly abundant in section IV of *K. sydneyanus* adults. Section V lumen was dominated by the genera *Rikenella* and *Alistipes* in all fish types, followed by *Faecalibacterium, Desulfovibrionaceae,* and *Akkermansia* (Fig. 5). In *K. sydneyanus* adults, the relative abundances of *Akkermansia* and *Alistipes* appeared to be influenced by temporal shifts, but not seasonal changes (see [11]). When samples were arranged chronologically (summer 2020 to autumn 2021), it was observed that the relative abundance of *Akkermansia* decreased while that of *Alistipes* increased in 2021. Estimated absolute abundance (16S rRNA copies/mL) in the lumen of all fish types increased from section III to section V and was greatest in adult individuals of *K. sydneyanus* section V (8.5 × 10^5^ ± 2.4×10^4^ mean copies/mL ± SE, Fig. 5 and Fig. S1).

Mucosa and lumen communities differed in terms of both the bacterial community abundance and structure of genera and 16S rRNA gene concentrations in all three groups, *K. sectatrix* and *K. sydneyanus* juveniles and adults, particularly in sections III and IV (lumen in Fig. 5 versus mucosa in Fig. 6). *Romboutsia,* followed by DMI, dominated samples of juvenile *K. sydneyanus* in sections III and IV, and *Desulfovibrionaceae* was highly abundant in some samples of section IV. *Lachnoclostridium* was the dominant genus in sections III and IV of *K. sectatrix*, with *Erysipelatoclostriudium* also being abundant in section IV. In *K. sydneyanus* adults, *Tyzzerella*, *Lachnoclostridium,* and DMI were the dominant genera and varied relative abundances across individual fish. Section V was notably dominated by *Treponema* and *Desulfovibrionaceae* along with *Alistipes*, *Rikenella,* and *Romboutsia* in both *K. sectatrix* adults and juveniles, especially juveniles, but with proportionally fewer *Alistipes* compared with the lumen. As in lumen samples of *K. sydneyanus* adults, the relative abundances of *Akkermansia* and *Alistipes* in the mucosa samples appeared to be influenced by a temporal shift. When *K. sydneyanus* samples were arranged chronologically (summer 2020 to autumn 2021) (Fig. 6), a decrease in the abundance of *Akkermansia* and an increase in the abundance of *Alistipes* were apparent in 2021. This temporal shift was not related to seasonal changes (see also reference 11). Estimated absolute abundances of bacteria in mucosa samples from *K. sectatrix* were highly variable (2.9 × 10^5^ ± 8.6 × 10^4^ copies/mL mean ± SE, in section III and 1.5 × 10^5^ ± 4.1 × 10^4^ copies/mL mean ± SE in section V) (Fig. S2). In *K. sydneyanus* juveniles, the estimated absolute abundances in sections III and IV were very low across all samples, with a slight increase in section V (~8.6 × 10^4^ ±1.9 × 10^4^ copies/mL mean ± SE). In *K. sydneyanus* adults, the estimated absolute abundances were also generally very low (3. × 10^4^ ± 4.6 × 10^3^ copies/mL mean ± SE in section III, 6.3 × 10^4^ ± 4.4 × 10^3^ copies/mL mean ± SE in section IV, 1.0 × 10^5^ ± 5.5 × 10^3^ copies/mL mean ± SE in section V, Fig. S2), except in some individuals with ≥5.0 × 10^8^ copies/mL across the three gut sections (Fig. 6). Overall, the estimated absolute abundances in all fish types compared were much lower in the mucosa (e.g., 6.4 × 10^3^ ± 1.6 × 10^3^ copies/mL mean ± SE in *K. sydneyanus* juveniles, Fig. 6; Fig. S2) than the lumen samples (3.4 × 10^4^ ± 1.1 × 10^4^ copies/mL mean ± SE, Fig. 5 and Fig. S1). This overall trend is consistent with most of the microbial fermentation in this fish species occurring primarily in the lumen of section V (11, 29).

**Fig 7:**
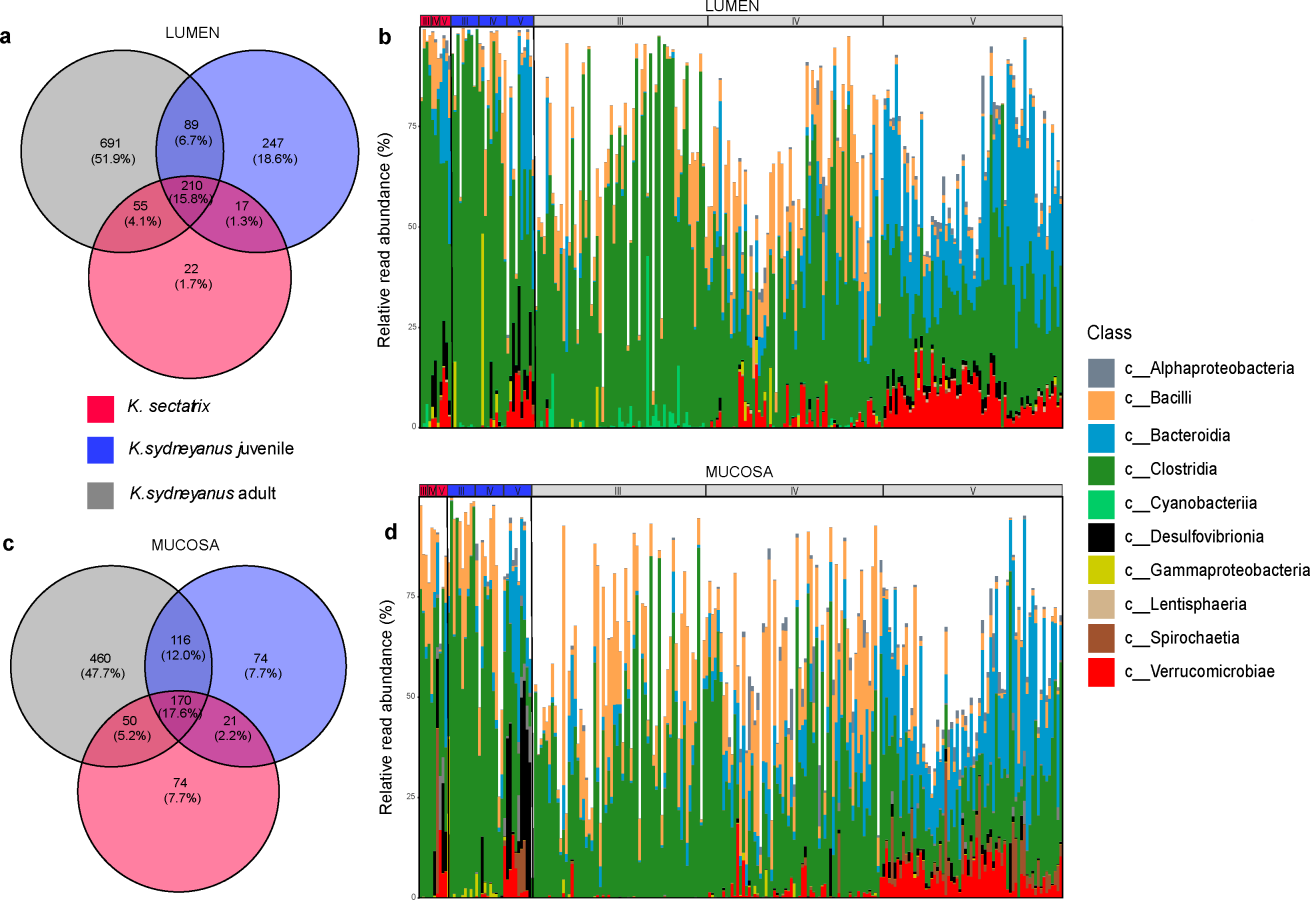
Shared ASVs and taxonomy among fish. (**a**) Venn diagram showing common taxa shared between *K. sectatrix*, *K. sydneyanus* juveniles, and *K. sydneyanus* adults in the lumen (sections III, IV, and V); (**b**) bar plots of the shared taxa between *K. sectatrix*, *K. sydneyanus* juveniles, and *K. sydneyanus* adults by class level for individual fish and gut sections. (**c**) and (**d**) are Venn diagram and bar plots for the mucosa sample.

A Venn diagram and bar plots of shared taxa at the class level were produced to compare the common taxa present between adult *K. sectatrix* and juvenile and adult *K. sydneyanus* (Fig. 7). In the lumen, 210 ASVs were shared between all fish species, 299 between adults and juvenile *K. sydneyanus*, 227 between *K. sectatrix* and juvenile *K. sydneyanus*, and 296 between *K. sectatrix* and adult *K. sydneyanus* (Fig. 7a). In the mucosa, there were 170 ASVs that were shared at the class level among all three sample types, 286 between adults and juvenile *K. sydneyanus*, 191 between *K. sectatrix* and juvenile *K. sydneyanus*, and 220 between *K. sectatrix* and adult *K. sydneyanus* (Fig. 7c). In both lumen and mucosa, the dominant common classes across all samples were *Clostridia* and *Bacilli* in sections III and IV and *Bacteroidia*, *Bacilli,* and *Verrucomicrobia* in section V (Fig. 7b and d). The low biomass in mucosal samples limits the interpretation of resident microbiota, as these samples may be subject to contamination from luminal populations. Further work is needed to confirm the exact compositions of lumen-associated communities.

### Association of habitat and community composition

**Fig 8:**
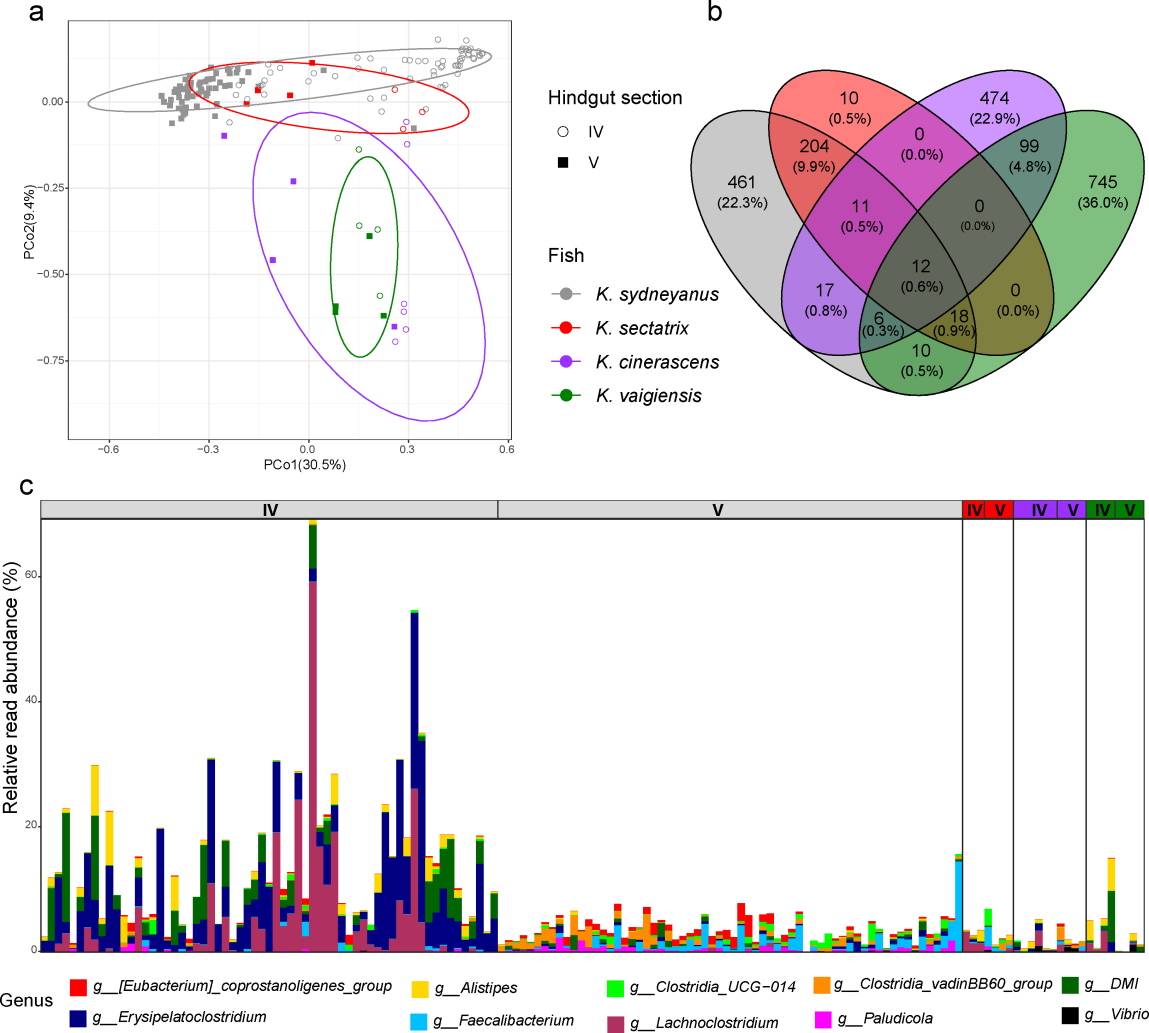
Summary figure of gut microbiota among all adult fish for lumen sections IV and V analyzed in this thesis: *K. sydneyanus* (gray), *K. sectatrix* (red), *K. cinerascens* (purple), and *K. vaigiensis* (green). (**a**) PCoA weighted UniFrac distance between fish. (**b**) A Venn diagram showing common taxa shared between fish. Circles represent shared ASVs between fish. (**c**) Bar plots of shared taxa (*n* = 12) between fish by genus level (*n* = 10) for individual fish and gut sections.

To further investigate community assembly of *Kyphosus* species, we compared the gut microbiota of adult *K. sydneyanus* and *K. sectatrix* collected in north-eastern New Zealand (temperate waters) with the gut microbiota of adult *K. cinerascens* and *K. vaigiensis* collected in the proximity of Lizard Island, Australian Great Barrier Reef (tropical waters). PCoA weighted UniFrac results indicate that *K. sydneyanus* and *K. sectatrix* have a more similar microbial composition when compared to the tropical *K. cinerascens* and *K. vaigiensis* (Fig. 8a). *K. sectatrix* has a very low percentage of novel taxa compared to the other *Kyphosus* species and shares a much higher number of ASVs with *K. sydneyanus* than with tropical *K. cinerascens* and *K. vaigiensis* (Fig. 8b). In terms of common ASVs, the four species only shared 12 ASVs (Fig. 8b) belonging to 10 genera (Fig. 8c).

## DISCUSSION

*Kyphosus* species are marine herbivores that rely on their gut microbiota to digest and assimilate dietary seaweeds (19, 20, 29, 30). Given the lack of vertical transmission of the gut microbiota of *Kyphosus* species, we sought to determine how multiple ecological drivers interact to assemble gut microbiota among wild populations. We found that microbial diversity is influenced by ontogeny and host species, and that variation in gut microbiota communities is strongly associated with diet. Historical contingency (the retention of early colonizing taxa) and environmental selection/dispersal limitation (the local pool of bacteria) play crucial roles in shaping the gut microbiota throughout *K. sydneyanus* development. The comparison of *Kyphosus* species from temperate New Zealand and tropical Australia highlights the role of dispersal limitation. Our results emphasize the importance of multiple ecological factors in shaping the gut microbiome in *Kyphosus* species.

### Diversity varies with ontogeny and species

In this study, beta and alpha diversity (Fig. 2 and 3) varied across the *K. sydneyanus* ontogenetic stage and across fish species. Gut microbial alpha diversity was highest in gut section V of adult individuals of *K. sydneyanus*, supporting the Sanger sequence-based results of (8). Diversity was also found to increase with fish age in *Silurus meridionalis* (31). The increased alpha diversity found in this study correlates with the higher concentration of SCFA found in adults than in juveniles (8). It is likely that the increased alpha diversity in the gut microbiota population with fish size may constitute a higher functional capacity (e.g., fermentation of phaeophyte carbohydrates, including mannitol and alginic acid) for unlocking more nutrients, which are otherwise refractory to adult fish. Therefore, the increasing alpha diversity of gut section V adult individuals of *K. sydneyanus* found in this study could reflect greater microbial fermentation activity, in addition to more robust and established microbial communities of adult fish. In ruminants, microbial diversity, colonization, and establishment of symbiotic microbes in the gut are affected by rumen development (32). It is possible that peak gut microbiota diversity and functionality are not reached until adulthood, when the gut is fully developed and gut retention times are longer. Retention time in subadult *K. sydneyanus* is ~21 hours (29), but it will be longer in the larger adults.

Alpha diversity was the highest in adult fish (Fig. 3). However, in terms of community structure and composition, some bacteria that were very abundant in the communities of *K. sydneyanus* adults (i.e., genus *Tyzzerella,* [11]) were much less abundant in *K. sectatrix* adults and *K. sydneyanus* juvenile individuals (Fig. 5 and 6), while other relatively abundant bacterial taxa were found across all three fish sample types. For example, community compositions of sections III and IV of the lumen and mucosa were dominated by *Bacillota* in all fish (Fig. 4), with *Romboutsia*, DMI, and *Lachnoclostridium* among the dominant genera. The proportion of these taxa was also high in the gut microbiota of previously analyzed *K. sydneyanus* adults (11, 19, 20). Together, this suggests that the ontogenetic change in diet was not associated with a drastic or complete change in the overall composition of gut microbial communities.

Section V represents the distal portion of the gut where SCFA levels and microbial biomass are highest in the lumen of *K. sydneyanus* adults (11, 29, 30). Similarly, the estimated absolute bacterial abundance increased from section III to V in the lumen and mucosa of *K. sydneyanus* juveniles, and in the lumen of *K. sectatrix*. The trend was the opposite in *K. sectatrix* mucosa, where abundances were highly variable across all sections. Further sampling of *K. sectatrix* individuals is needed to confirm this difference in the mucosa. Overall, section V showed the highest variability in community composition between fish in both lumen and mucosa samples (Fig. 4). As the three fish sample types were collected from the same locations in New Zealand, these findings suggest that historical contingency and environmental selection based on local variations in factors, such as time, diet, and fish species, likely influence the dynamics of bacteria colonizing the gut of these fish. It is likely that community composition in adult fish involves a combination of founding bacterial taxa that colonized fish as juveniles and later introductions due to the ongoing colonization of adults. The retention of both early and later colonizing taxa, as suggested by our identification of shared taxa between juveniles and adults (Fig. 7), helps to explain why microbial diversity is higher in adults.

In the Atlantic salmon, the ontogenetic development of the fish gut microbiota corresponded to a shift in the dominant hindgut microbiota from *Pseudomonadota* to *Bacteroidota* and *Bacillota* (5). In this study, *Bacillota* and *Bacteroidota* instead dominated the microbial composition of both juveniles and adults of *K. sydneyanus*, with *Bacillota* dominating sections III and IV of both lumen and mucosa and *Bacteroidota* co-dominating in section V. Moreover, taxa from the *Bacteroidota* genera *Alistipes* and *Rikenella* were some of the most abundant and common taxa in *K. sydneyanus* adults, and their abundance was similarly high in *Kyphosus* juveniles (10, 11, 19, 20). The high abundance of *Bacteroidota* is suggested to favor rumen development in calves (33) and may play a similar role in *Kyphosus* with respect to gut development.

### Relationship between diet, microbial composition, and ontogeny

Our *K. sectatrix* had a high proportion of Rhodophytes in its stomach (Fig. 1), similar to that reported from *K. sectatrix* collected in the Caribbean Sea (27) and the southwest Atlantic Ocean (26). The *K. sectatrix* analyzed in reference 28 (referred to as *K. bigibbus* therein [24]) were 235–350 mm standard length (SL), while those sampled in reference 26 were 199–392 mm fork length (FL, which is the measure from the most anterior part of the fish to the fork/end of the caudal fin). Overall, the *K. sectatrix* collected for the present study (i.e., 270–305 mm standard length) were somewhat smaller than those in (28), although they were sampled from the same locations. This could suggest that our *K. sectatrix* samples were sub-adults, and this species may also undergo a similar ontogenetic dietary shift to *K. sydneyanus* from predominantly Rhodophyta in juveniles to predominantly Phaeophyceae in adults. This would be consistent with the similarities in gut anatomy between *K. sectatrix* and *K. sydneyanus* (Fig. 4). However, this hypothesis has yet to be tested by better sampling of diet in both juvenile and adult size classes of *K. sectatrix*, and comparisons between *K. sydneyanus* juveniles and *K. sectatrix* as a distinct species are still valid for testing the relative influence of diet and ontogeny.

Diet is one of the main drivers of gut microbiota composition in fish (7, 17, 34). When we considered taxa common across fish in terms of their community relative abundance (Fig. 7b and d), *K. sectatrix* and *K. sydneyanus* juveniles both harbored the same very high proportion of *Clostridia* in their gut communities compared with adults of *K. sydneyanus*. This suggests that the similar feeding choices of *K. sectatrix* and *K. sydneyanus* juveniles influence gut microbiota community assembly in these fish. As illustrated in Fig. 5, *Romboutsia* (within the *Clostridia*) is consistently very high in the lumen of both *K. sectatrix* and *K. sydneyanus* juveniles in sections III and IV (and higher in V) compared to adults of *K. sydneyanus*. The genus *Romboutsia* is typically found in the gut of mammals (35), and little is known about their specific function. However, a recent study on the comparative and functional genomics analysis showed that members of the genus *Romboutsia,* isolated from intestinal and environmental samples, possess a range of metabolic capabilities, including carbohydrate utilization, amino acid fermentation, and anaerobic respiration (35). Moreover, *Romboutsia* was positively associated with total carbohydrate digestibility in a piglet dietary study (36). Our results are constrained in functional interpretation by the use of ASV-based taxonomic assignments, which do not directly reflect microbial function, and interpretation of function is further constrained in less well-studied non-human systems. However, the relative abundance and distribution of *Romboutsia* in our study suggest that they may play an important role in carbohydrate utilization and nutrient assimilation from dietary algae in *K. sectatrix* and *K. sydneyanus* juveniles. In contrast, *K. sectatrix* community compositions when considering shared ASVs did not differ substantially from those of *K. sydneyanus* adults (Fig. 7a and c). These findings suggest that, as the diet of *K. sectatrix* could sometimes be dominated by phaeophytes (28), they may need a gut microbiota able to exploit nutrients from a variety of algal taxa.

### Historical contingency and environment contribute to the microbial composition of adults

Adults and juveniles of *K. sydneyanus* shared the highest number of common ASVs, especially in the lumen, and a high proportion of *Bacteroidia* relative abundance (section V, both lumen and mucosa, Fig. 7). This could suggest that the juvenile gut microbial communities are a subset of adult fish communities. The high level of shared ASVs among the three sample types in our study could suggest that the environment/habitat where the fish live plays a major role in shaping the gut microbiota of our fish (which were all collected from similar locations). Historical contingency (fish development) and dispersal limitation (the bacteria present in the local environment) (6) may play key roles in how these taxa established in the gut of *K. sydneyanus* species. The gut environment may also select for the microbial communities in the gut of fish during its development (3). In some fish species (e.g., sturgeon), it is likely that during early ontogenetic periods, host development has a deterministic effect on the formation of gut communities regardless of dietary sources or surrounding environment (4).

To further investigate the processes influencing community assembly processes in *Kyphosus* species, we compared the adult gut microbiota of *Kyphosus* species analyzed in references 10, 11 (Fig. 8). Results based on comparisons of *K. sydneyanus, K. sectatrix, K. cinerascens,* and *K. vaigiensis* were consistent with our hypothesis that dispersal limitation and selection play a major role in shaping the gut community. Fish collected from the same location (temperate *K. sydneyanus* and tropical *K. sectatrix* from north-eastern New Zealand versus tropical *K. cinerascens* and *K. vaigiensis* from the Australian Great Barrier Reef) shared a much higher number of ASVs and much more similar microbial community compositions (Fig. 8), consistent with dispersal limitation along with selection based on local environmental factors. Moreover, only one out of the 12 shared ASVs belonged to a genus (i.e., *Alistipes*) that was also found in *Kyphosus* from other locations (Hawaii [37]). The two locations, northeastern New Zealand (temperate) and the Australian Great Barrier Reef (tropical), differ greatly in their environments and available algal food resources. New Zealand temperate habitats are typically dominated by kelps such as *E. radiata* that constitute the bulk of the diet of *K. sydneyanus* in New Zealand (Fig. 1) (25). In contrast, the Great Barrier Reef in Australia is characterized by tropical algae that constitute the diet of *Kyphosus* species living in those areas, including *Laurencia majuscula*, *Sphacelaria tribuloides,* and *Cladophora rugulosa* for *Kyphosus cinerascens*, and *Udotea argentea* and *Turbinaria ornata* for *K. vaigiensis* (38). This suggests that, regardless of diet and environment (tropical or temperate), the pool of bacteria present in the environment where the fish live is key in gut community assembly. Dispersal limitation is also suggested to drive community assembly in other fish (4, 5), mammals (39), and fungal species (40).

### Conclusions

In conclusion, although this study is limited by (i) the use of ASVs to provide taxonomic assignments of microbiota, and thus lacks direct functional data, and (ii) uneven sampling due to the low abundance of *K. sectatrix* in New Zealand, it presents novel data, including interspecific comparisons among *Kyphosus* species from distinct environments (temperate New Zealand and tropical Australia). It also incorporates both relative and absolute abundance estimates of bacterial taxa generated using high-throughput sequencing and droplet digital PCR (ddPCR). To the extent of our knowledge, only one previous study has investigated the gut microbiota of *K. sydneyanus* juveniles (8). While this previous study provided important initial findings on ontogenetic changes in microbial diversity, it was limited by techniques yielding relatively low numbers of sequences and no quantitative data on estimated bacterial abundance. *K. sydneyanus* juveniles shared a higher number of ASVs with adult conspecifics who differ in diet (i.e., a high proportion of phaeophytes) than with adult *K. sectatrix* with a diet similar to juvenile *K. sydneyanus*. This underlines the importance of historical contingency in the community assembly process, that is, selected bacterial species present in the environment colonize and become established members of the gut microbiota in young fish. When looking at the community assembly process at a larger spatial scale (fish collected in northeast New Zealand versus fish collected from tropical coral reefs in Australia), dispersal limitation and environmental selection seem likely to be the main drivers of gut microbiota community assembly. Future work should compare the gut microbiota of these fish with the microbial communities of other herbivorous fish species that feed on different dietary sources. This would improve our understanding of community assembly processes and the factors influencing the gut microbial composition in wild marine herbivorous fishes. Future research could also explore the functional potential of bacterial taxa found across juvenile and adult fish in order to determine how the capacity to degrade polysaccharides from different algal taxa develops in relation to ontogenetic changes in diet.

## MATERIALS AND METHODS

### Sample collection and data set composition

*K. sydneyanus* juveniles (*n* = 10) and *K. sectatrix* adults (*n* = 4) were collected by spear gun on snorkel from different locations around Great Barrier Island (36˚17ʹ S, 175˚20ʹ E), Mokohinau Islands (35˚55ʹ, 175˚08ʹ), Double Island (36˚37ʹ S 175˚54ʹ E), and Cuvier Island (36˚25ʹ S, 175˚46ʹ E), New Zealand from 2017 to 2022. Sample size for *K. sectatrix* was constrained due to its limited occurrence in New Zealand waters (23, 24). For each fish, one sample of lumen and one sample of mucosa were collected from hindgut sections III, IV, and V following references 11 and 38, generating six samples per fish individual and a total of 84 gut section samples (details of specimens and collection sites are in Table S1). To collect mucosal samples, following the collection of lumen content, the intestine was slit longitudinally and the gut walls carefully rinsed with Milli-Q water to remove luminal content. Mucosal samples were collected using a spatula following references 11 and 20. Stomach content was photographed at the point of capture to allow direct comparisons of diet with gut microbiota composition. Additional samples included in the study were as follows: hindgut section III, IV, and V, lumen and mucosa samples from adult *K. sydneyanus* (*n* = 64 fish, *n* = 378 lumen and mucosa gut sections) from our previous publication (National Center For Biotechnology Information [NCBI]: https://www.ncbi.nlm.nih.gov/bioproject/PRJNA930464/; Accession: PRJNA930464) (11), samples of *Ecklonia radiata* (*n* = 10 specimens) from European Nucleotide Archive (ENA): https://www.ebi.ac.uk/ena/browser/view/PRJEB34792; Accession: PRJEB34792 (19) and stomach content replicates (*n* = 2) from a *K. sydneyanus* adult (Table S1). Samples were immediately snap-frozen in liquid nitrogen and transported to the University of Auckland for analysis. Hindgut lumen samples from sections IV and V of *K. cinerascens* (*n* = 6 fish, *n* = 10 lumen gut sections) and *K. vaigiensis* (*n* = 4 fish, *n* = 8 lumen gut sections), collected in 2017 by spear gun while snorkeling around the Lizard Island complex, Great Barrier Reef, Australia (14 40ʹ S, 145 28ʹ E) (samples recovered from National Center For Biotechnology Information (NCBI): https://www.ncbi.nlm.nih.gov/bioproject/?term=PRJNA783643; Accession: PRJNA783643 [10]) (Table S1) were successively added to the analysis for additional comparisons. Fish collection was conducted under University of Auckland Animal Ethics Committee approvals 001636, 001949, and 2879. Length and weight were recorded. *K. sydneyanus* juveniles collected ranged from 165 to 237 mm standard length (SL, which is the measure from the most anterior part of the fish to the base of the caudal fin), corresponding to immature fish ~1–4 years of age (25). *K. sectatrix* collected were 270–305 mm standard length (Table S1).

### DNA extraction, amplicon sequencing, and quantification

DNA extraction, PCR amplification, and sequencing were performed as described in reference 11. In brief, DNA was extracted from samples using the DNeasy PowerSoil DNA Isolation Kit (Qiagen, Hilden, Germany) with ~250 mg of material. Extracted DNA was quality and quantity checked using a NanoPhotometer (Implen, Germany) and Qubit 3.0 (Thermo Fisher Scientific, Waltham, MA, USA). PCR amplification of the 16S rRNA gene was performed following reference 11 using the bacterial primers 341F/785R (5ʹ-CCTACGGGNGGCWGCAG-3ʹ and 5ʹ-GACTACHVGGGTATCTAATCC-3ʹ) to target the V3-V4 region (41, 42) with Illumina overhang adapters (Document # 15044223 Rev. B, Illumina, USA). PCR products were purified using Agencourt AMPure XP magnetic beads (Beckman Coulter, CA, USA). Barcoded library preparation and 2 × 300 bp paired-end Illumina MiSeq sequencing were performed with the V3 MiSeq reagent kit by Auckland Genomics (University of Auckland, NZ). Quantification of 16S rRNA gene copies was performed using droplet digital PCR (ddPCR) to generate estimated absolute read abundances. Conditions and contamination check for ddPCR were described in reference 11. In brief, ddPCR primers were the same as used for PCR of 16S rRNA gene amplicons but without Illumina adapters, and reactions were carried out using 2× QX299 ddPCR EvaGreen supermix (Bio-Rad Laboratories, CA, US) and to exclude samples and background contamination three negative controls (no template) were included in each run, and the average of those negatives was deducted from the samples (11). To generate estimated absolute abundance for 16S rRNA gene amplicon sequence data, ASV proportions per sample (generated as described below) were multiplied by ddPCR values, estimated values of bacterial density expressed in copies/mL (11).

### Bacterial microbiota analysis

16S rRNA gene amplicon sequences were analyzed with QIIME2 (v 2021.2) (43). All sequences from previously published studies added to the analyses were from our group, and they all were consistent with the methodology outlined in this study, including DNA extraction and amplification of the V3-V4 rRNA region (10, 11, 19). Demultiplexed sequences were read-joined, quality filtered (Q score cutoff of 25, poor quality samples were discarded), and denoised (singletons removal) (44) using QIIME2 with the Deblur plugin (45) to generate ASVs. ASVs were taxonomically classified using the SILVA database v138 (46–48) with phyla taxonomy updated to the new NCBI phylum taxonomy (49). QIIME2 was also used to rarefy the ASV table at 1052 reads per sample (43), leaving a total of 2,206 ASVs across the samples. This approach was used to retain most of the samples and avoid losing valuable biological replicate data, particularly given the uneven sampling in our data set. Data visualization was performed using R v4.2.1 with RStudio software version 1.2.1335 and the R package ggplot2 v3.3.5. Statistical analysis was executed with the adonis2 function (vegan v2.5-7) for permutational multivariate analysis of variance (PERMANOVA) and Kruskal-Wallis with Dunn post-hoc (dunn.test v1.3.5) (50).

## Data Availability

The raw reads analyzed in the current study have been deposited at the National Center for Biotechnology Information (NCBI, https://www.ncbi.nlm.nih.gov/) and are available under the BioProject IDs PRJNA930464 and PRJNA783643. *E. radiata* raw sequences are available at the European Nucleotide Archive (ENA, https://www.ebi.ac.uk/ena/browser/home) PRJEB34792.

## SUPPLEMENTAL MATERIAL

Supplemental material spectrum.02317-25-s0001.pdf Tables S2 to S6; Fig. S1 and S2.

Table S1 spectrum.02317-25-s0002.xlsb Details of sample collection.

ASM does not own the copyrights to Supplemental Material that may be linked to, or accessed through, an article. The authors have granted ASM a non-exclusive, world-wide license to publish the Supplemental Material files. Please contact the corresponding author directly for reuse.
