# Supplementary material for: Ontogenetic variation in the gut microbiota of Kyphosus sydneyanus: a comparative analysis
Source: Microbiol Spectr. 2025 Oct 20;13(12):e02317-25. doi: 10.1128/spectrum.02317-25 (PMC12671223; doi:10.1128/spectrum.02317-25)
Supplement: Supplemental material — Tables S2 to S6; Fig. S1 and S2. [file spectrum.02317-25-s0001.pdf]

Supplementary Table 2. Statistical analysis for beta diversity measures (weighted UniFrac and Bray-Curtis) tested using PERMANOVA.

**Weighted UniFrac matrix**		Df	Sum of Sqs	R2	F	Pr(>F)
Lumen	Fish/*E. radiata* × section	4	0.510	0.01533	2.4753	0.005
	Fish/*E. radiata*	3	6.754	0.02302	43.6941	0.001
	Hindgut sections (III, IV, V)	2	14.154	0.42544	137.3525	0.001
Mucosa	Fish/*E. radiata* × section	4	0.529	0.1652	2.5096	0.001
	Fish/*E. radiata*	3	7.004	0.21879	44.3280	0.001
	Hindgut sections (III, IV, V)	2	12.418	0.38792	117.8901	0.001
**Bray-Curtis matrix**		**Df**	**Sum of Sqs**	**R2**	**F**	**Pr(>F)**
Lumen	Fish/*E. radiata* × section	4	3.526	0.03662	3.2134	0.001
	Fish/*E. radiata*	3	13.538	0.14062	16.4512	0.001
	Hindgut sections (III, IV, V)	2	16.122	0.16746	29.3878	0.001
Mucosa	Fish/*E. radiata* × section	4	3.308	0.03499	2.9554	0.001
	Fish/*E. radiata*	3	13.205	0.13966	15.7275	0.001
	Hindgut sections (III, IV, V)	2	13.948	0.14752	24.9185	0.001

Supplementary Table 3. Statistical analysis for alpha diversity (Shannon diversity index) comparing gut sections in lumen and mucosa for each fish using Kruskal-Wallis with post-hoc Dunn test.

Gut site	Fish	chi2	Z	P	P. adjusted	Comparisons (n=3)
Lumen	K. sectatrix	8.909	-1.382	0.084	0.084	III - IV
Lumen	K. sectatrix	8.909	-2.985	0.001	0.004**	III - V
Lumen	K. sectatrix	8.909	-1.382	0.084	0.125	IV - V
Mucosa	K. sectatrix	0.118	0.270	0.394	0.591	III - IV
Mucosa	K. sectatrix	0.118	-0.036	0.486	0.486	III - V
Mucosa	K. sectatrix	0.118	-0.324	0.373	1.000	IV - V
Lumen	K. sydneyanus adult	114.565	-5.106	0.000	0.000***	III - IV
Lumen	K. sydneyanus adult	114.565	-10.698	0.000	0.000***	III - V
Lumen	K. sydneyanus adult	114.565	-5.594	0.000	0.000***	IV - V
Mucosa	K. sydneyanus adult	93.657	-3.345	0.000	0.000***	III - IV
Mucosa	K. sydneyanus adult	93.657	-9.526	0.000	0.000***	III - V
Mucosa	K. sydneyanus adult	93.657	-6.192	0.000	0.000***	IV - V
Lumen	K. sydneyanus juvenile	12.924	-1.321	0.093	0.093	III - IV
Lumen	K. sydneyanus juvenile	12.924	-3.556	0.000	0.001***	III - V
Lumen	K. sydneyanus juvenile	12.924	-2.235	0.013	0.019*	IV - V
Mucosa	K. sydneyanus juvenile	7.714	-1.575	0.058	0.086	III - IV
Mucosa	K. sydneyanus juvenile	7.714	-2.769	0.003	0.008**	III - V
Mucosa	K. sydneyanus juvenile	7.714	-1.194	0.116	0.116	IV - V

Supplementary Table 4. Statistical analysis for alpha diversity (Shannon diversity index) comparing fish in lumen and mucosa for each gut section using Kruskal-Wallis with post-hoc Dunn test.

Gut site	Section	chi2	Z	P	P. adjusted	Comparisons (n=3)
Lumen	III	11.005	-2.083	0.019	0.028*	K. sec - K. syd adu
Lumen	III	11.005	-0.226	0.411	0.411*	K. sect - K. syd juv
Lumen	III	11.005	2.762	0.003	0.009**	K. syd adult - K. syd juv
Lumen	V	17.748	-0.780	0.218	0.218	K. sec - K. syd adu

Lumen	V	17.748	1.731	0.042	0.063	K. sec - K. syd juv
Lumen	V	17.748	4.193	0.000	0.000***	K. syd adu - K. syd juv
Mucosa	V	21.631	-2.533	0.006	0.008**	K. sec - K. syd adu
Mucosa	V	21.631	0.157	0.438	0.438	K. sect - K. syd juv
Mucosa	V	21.631	4.111	0.000	0.000***	K. syd adu - K. syd juv
Mucosa	III	15.132	0.559	0.288	0.288	K. sec - K. syd adu
Mucosa	III	15.132	2.465	0.007	0.010*	K. sec - K. syd juv
Mucosa	III	15.132	3.793	0.000	0.000***	K. syd adu - K. syd juv
Lumen	IV	18.669	-1.355	0.088	0.132	K. sec - K. syd adu
Lumen	IV	18.669	0.954	0.170	0.170	K. sec - K. syd juv
Lumen	IV	18.669	4.197	0.000	0.000***	K. syd adult - K. syd juv
Mucosa	IV	9.134	-0.937	0.174	0.262	K. sec - K. syd adu
Mucosa	IV	9.134	0.679	0.249	0.249	K. sec - K. syd juv
Mucosa	IV	9.134	2.938	0.002	0.005**	K. syd adu - K. syd juv

Supplementary Table 5. Statistical analysis for alpha diversity (ASVs richness index) comparing gut sections in lumen and mucosa for each fish using Kruskal-Wallis with post-hoc Dunn test.

**Gut site**	**Fish**	**chi2**	**Z**	**P**	**P. adjusted**	**Comparisons (n=3)**
Lumen	K. sectatrix	8.364	-1.154	0.124	0.124	III - IV
Lumen	K. sectatrix	8.364	-2.885	0.002	0.006**	III - V
Lumen	K. sectatrix	8.364	-1.517	0.065	0.097	IV - V
Mucosa	K. sectatrix	0.869	0.676	0.249	0.374	III - IV
Mucosa	K. sectatrix	0.869	-0.181	0.428	0.428	III - V
Mucosa	K. sectatrix	0.869	-0.904	0.183	0.549	IV - V
Lumen	K. sydneyanus adult	110.732	-4.677	0.000	0.000***	III - IV
Lumen	K. sydneyanus adult	110.732	-10.497	0.000	0.000***	III - V
Lumen	K. sydneyanus adult	110.732	-5.825	0.000	0.000***	IV - V
Mucosa	K. sydneyanus adult	115.533	-4.595	0.000	0.000***	III - IV
Mucosa	K. sydneyanus adult	115.533	-10.706	0.000	0.000***	III - V
Mucosa	K. sydneyanus adult	115.533	-6.119	0.000	0.000***	IV - V
Lumen	K. sydneyanus juvenile	8.336	-1.843	0.033	0.049*	III - IV
Lumen	K. sydneyanus juvenile	8.336	-2.846	0.002	0.007**	III - V
Lumen	K. sydneyanus juvenile	8.336	-1.004	0.158	0.158	IV - V
Mucosa	K. sydneyanus juvenile	13.368	-2.681	0.004	0.006**	III - IV
Mucosa	K. sydneyanus juvenile	13.368	-3.494	0.000	0.001***	III - V
Mucosa	K. sydneyanus juvenile	13.368	-0.813	0.208	0.208	IV - V

Supplementary Table 6. Statistical analysis for alpha diversity (ASVs richness index) comparing fish in lumen and mucosa for each gut section using Kruskal-Wallis with post-hoc Dunn test.

**Gut site**	**Section**	**chi2**	**Z**	**P**	**P. adjusted**	**Comparisons (n=3)**
Lumen	III	3.106	-1.365	0.086	0.258	K. sect - K. syd adu
Lumen	III	3.106	-0.479	0.316	0.316	K. sectatrix - K. syd juv
Lumen	III	3.106	1.235	0.108	0.163	K. syd adu - K. syd juv
Lumen	V	23.577	-1.295	0.098	0.098	K. sec- K. syd adu
Lumen	V	23.577	1.617	0.053	0.079	K. sec - K. syd juv
Lumen	V	23.577	4.777	0.000	0.000***	K. syd adu - K. syd juv
Mucosa	V	22.228	-2.486	0.006	0.010**	K. sec - K. syd adu
Mucosa	V	22.228	0.256	0.399	0.399	K. sec - K. syd juv

Mucosa	V	22.228	4.212	0.000	0.000***	K. syd adu - K. syd juv
Mucosa	III	6.698	1.171	0.121	0.121	K. sec - K. syd adu
Mucosa	III	6.698	2.194	0.014	0.021*	K. sec - K. syd juv
Mucosa	III	6.698	2.207	0.014	0.041*	K. syd adu - K. syd juv
Lumen	IV	4.252	-0.203	0.420	0.420	K. sec - K. syd adu
Lumen	IV	4.252	0.884	0.188	0.282	K. sec - K. syd juv
Lumen	IV	4.252	2.062	0.020	0.059	K. syd adu - K. syd juv
Mucosa	IV	5.198	-1.626	0.052	0.078	K. sec - K. syd adu
Mucosa	IV	5.198	-0.569	0.285	0.285	K. sec - K. syd juv
Mucosa	IV	5.198	1.722	0.043	0.128	K. syd adu - K. syd juv

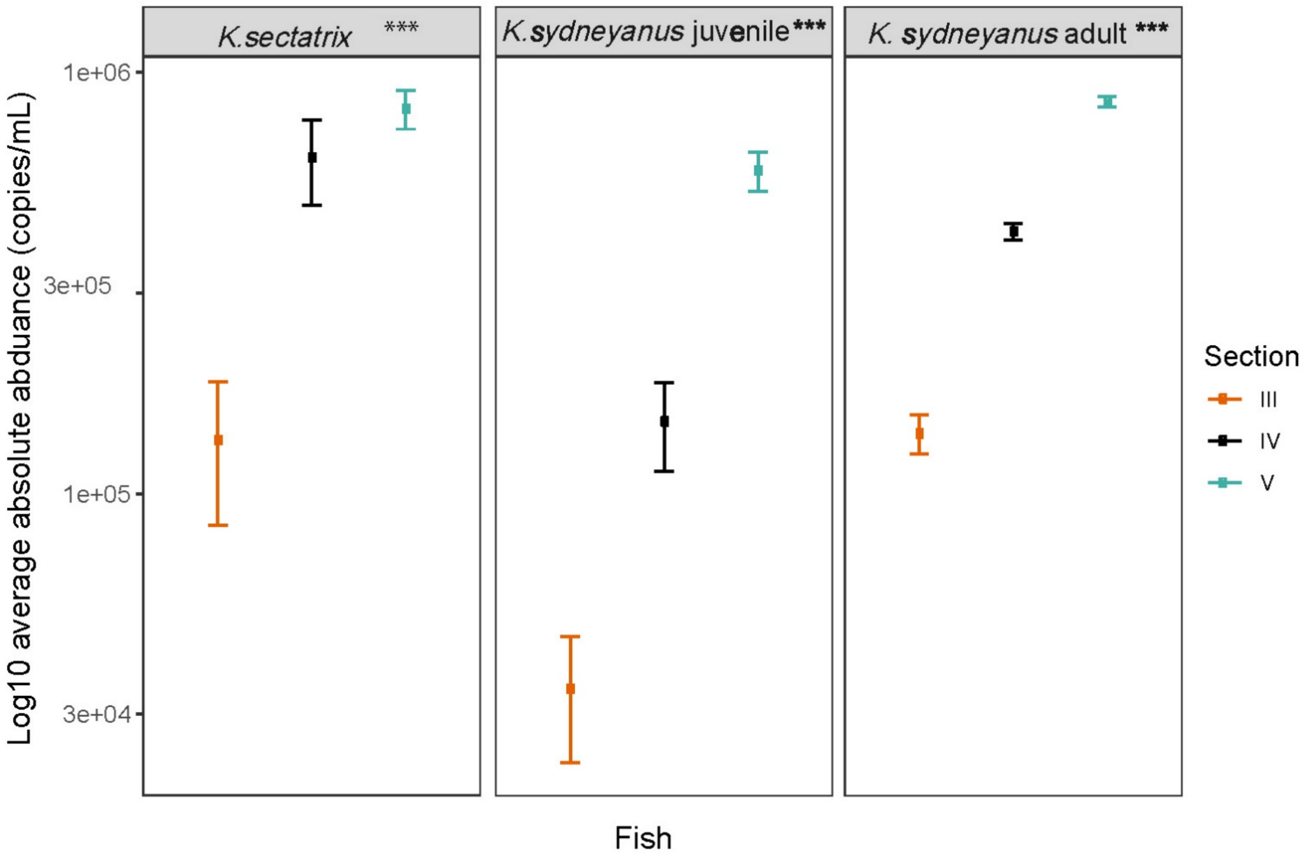

Supplementary Figure 1. Average of estimated absolute read abundance (copies/mL) in Log10 scale across gut sections in the lumen of *K. sectatrix*, *K. sydneyanus* juveniles and *K. sydneyanus* adults. Statistically significant differences between gut sections were tested using ANOVA test. Significance codes: 0 ‘***’ 0.001 ‘**’ 0.01 ‘*’ 0.05 ‘.’ 0.1 ‘ ’ 1.

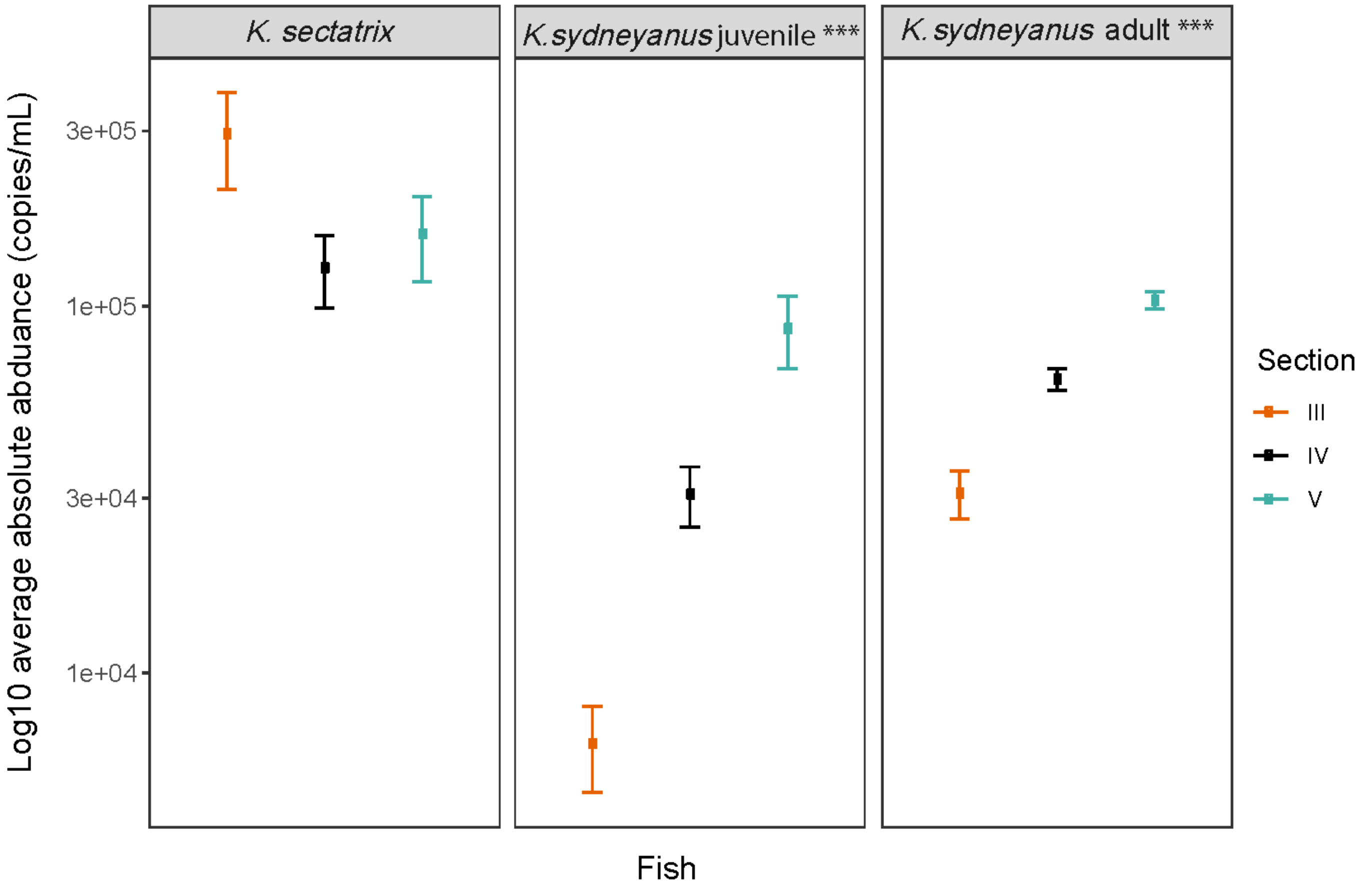

Supplementary Figure 2. Average of estimated absolute read abundance (copies/mL) in Log10 scale across gut sections in the mucosa of *K. sectatrix*, *K. sydneyanus* juveniles and *K. sydneyanus* adults. Statistically significant differences between gut sections were tested using ANOVA test. Significance codes: 0 '***' 0.001 '**' 0.01 '*' 0.05 '.' 0.1 ' ' 1.
